# Effectiveness of a self-management mobile app on the quality of life of women with breast cancer: a study in a developing country

**DOI:** 10.1186/s12905-022-02020-5

**Published:** 2022-11-11

**Authors:** Zahra Mohammadzadeh, Samereh Eghtedar, Haleh Ayatollahi, Mohamad Jebraeily

**Affiliations:** 1 Department of Health Information Technology, School of Allied Medical Sciences, Urmia University of Medical Sciences, Urmia, Iran; 2 Department of Health Information Management and Technology, School of Allied Medical Sciences, Kashan University of Medical Sciences, Kashan, Iran; 3 Department of Nursing Education, School of Nursing and Midwifery, Urmia University of Medical Sciences, Urmia, Iran; 4 Department of Obstetrics and Gynecology, School of Medicine, Solid Tumor Research Center, Urmia University of Medical Sciences, Urmia, Iran

**Keywords:** Breast Cancer, Self-management, Mobile app, Quality of life

## Abstract

**Background:**

Self-management involves taking responsibility for personal health and taking the initiative to do so. This can be accomplished by learning information and skills that will help consider the difficulties may encounter during and after cancer treatment. With this perspective, we have aimed to develop a self-management mobile app for women with breast cancer in Iran and evaluate its impact on the quality of life of patients.

**Method:**

This study is a methodological study in 2021. We developed the app during three phases. The first phase aimed at identifying educational content and designing user experience, the second phase aimed at developing and implementing the app, and the third phase aimed at evaluating pre-and post-implementation.

**Result:**

In this study, an Android app for self-management women with breast cancer was developed. According to the results of the pre-and post-implementations among the most significant changes were in the quality of life level, highest respectively: social avoidance (Pre: 6.41–Post: 3.56), negative feelings (Pre: 5.93 - Post: 3.40), sexual function (Pre: 6.80 - Post: 5.04), sexual interest (Pre: 6.41 - Post: 4.75) and pain (Pre: 6.37 - Post: 4.97). And least the changes respectively: distress-family (Pre: 7 - Post: 7), distress-recurrence (Pre: 4.49 - Post: 4.38), benefits (Pre: 2.47 - Post: 3.12), appearance (Pre: 4.10 - Post: 3.32). Also, we calculated the usability evaluation of this app with the system usability scale (SUS); the overall rating score was 83/100, an excellent level (> 80.3), and a grade A.

**Conclusion:**

The study shows that a breast cancer self-management app can support and improve the quality of life of women with breast cancer. We conducted this study to show that by developing a self-management app, women with breast cancer can improve their quality of life, − by increasing their self-management skills.

**Supplementary Information:**

The online version contains supplementary material available at 10.1186/s12905-022-02020-5.

## Introduction

The most common type of cancer in women and the one with the highest mortality rate is breast cancer [1]. A breast cancer diagnosis can be an unpleasant and unimaginable experience for any woman; it can disrupt her whole life [2]. The incidence of breast cancer dramatically enhanced in most developing countries, including Iran [3]. In low- or middle-income countries, lifestyle changes like obesity, dietary, and smoking patterns have a great implementation impact on breast cancer rates [4]; over 55% of breast cancers are ending in death in these countries [5]. According to a World Health Organization 2018 report, the estimated number of recently diagnosed breast cancer cases overall was over two million people around the world [6], − the highest among women’s cancers. More than 12,000 women are diagnosed to have breast cancer every year in Iran [7]. The peak age of its pervasiveness in Iranian women is identified as the fourth and fifth decades of life; notice that this is one decade lower than the age of its worldwide prevalence [8].

Breast cancer and its treatment have a huge effect on the psychological, social, emotional, and physical well-being of women with breast cancer [9]. The breast cancer treatment plan includes surgery, chemotherapy, radiation therapy, and hormonal therapy based on the stage of a cancer diagnosis for each patient [10]. In addition, breast cancer therapies have been linked with physical side effects such as a feeling of weakness and fatigue, appetite loss, hair loss, sore mouth, vomiting and nausea, and infertility [11, 12]. Despite the beneficial effects of treatment methods there by increasing patient survival, various psychosocial aspects, for example, stress, depression, indecision about the future, and worry of cancer recurrence and role of the patient in society are among the concerns of women with breast cancer [13, 14].

To live well with breast cancer, women must be able to manage their symptoms independently of their doctor. Indeed breast cancer is regarded currently as a chronic disease. In chronic disease management, self-management is one of the emerging operational strategies [15]. Kim et al. have found that self-management leads to symptom management, lifestyle management, and the promotion of patient health [16]. In breast cancer patients, self-management strategies include awareness about disease conditions, increased motivation, and self-efficacy [17]. When patients are more aware of the way and skills of the self-management of breast cancer, they are better able to handle their illness [16, 17]. Therefore, self-management apps could help patients better manage their health and change their behavior to improve health [18].

The Global Health Observatory (GHO) defines m-Health (mobile health) as “*medical and public health practice supported by mobile devices, such as mobile phones, patient monitoring devices, personal digital assistants, and other wireless devices*” [19]. In fact, m-Health is fundamental to the eventual fate of illness and health management [20]. The results of several studies have shown that using mobile apps to help patients with breast cancer has numerous benefits, including improved information needs, increased physical activity, decreased nervousness and anxiety, developed fearlessness, and improved personal satisfaction [21, 22].

In Iran, no study has yet investigated the impact of the use of mobile apps on the quality of life of breast cancer patients. However, a randomized controlled trial in Iran demonstrated that psychoeducational interventions through mobile apps could be helpful in decreasing anxiety and improving self-esteem in women with breast cancer [23]. As regards to Iran, which is considered as a developing country, the smartphone penetration rate is increasing and nearly 70% of the population in Iran have tablets or smartphones [24, 25]. In light of these existing contexts, women’s urgent need for self-management, and the availability of effective mobile apps, we aim to design, develop, and evaluate a mobile self-management app for Iranian women with breast cancer.

## Methods

**Fig. 1:**
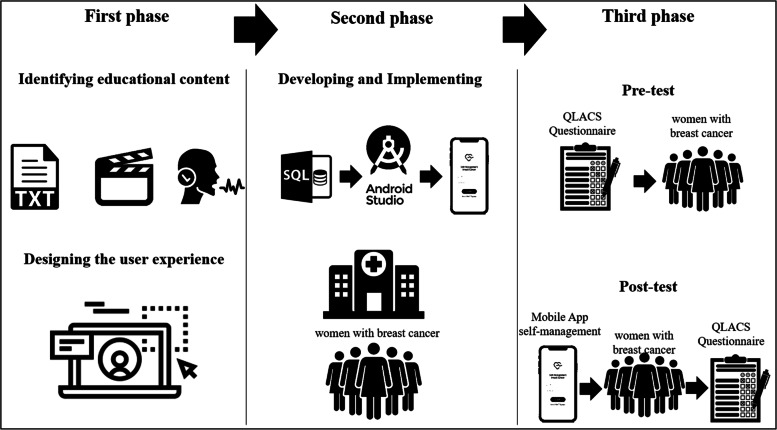
The first phase of research focused on determining the content for the app; this phase was completed with Balsamiq Mockups software to create the user experience; the second phase used an SQL server to develop the database and android studio for the development of the app; for this phase, we considered a number of women with breast cancer (*n* = 24). In the third phase, we conducted pre- and post-implementation using the questionnaire QLACS and the app developed

This study aimed to develop a mobile app for self-management for Iranian women diagnosed with breast cancer. To achieve this aim, we conducted our research in three phases. The first phase was about identifying educational content and designing user experience, the second phase was about developing and implementing the app, and the third phase evaluating was about pre-and post-implementation. We illustrate by a graphical abstract the research phases, in Fig. 1.

### First phase

We conducted a descriptive cross-sectional study of woman’s breast cancer patients in 2020 to identify the educational content used in the app [26]. As a matter of fact, sampling randomly selected 120 women with breast cancer in phase one of the study. Based on valid scientific texts and related articles, we analyzed educational content and technical features of the smartphone app. There are five main categories of educational contents, including information acquisition (7 items), lifestyle management (7 items), psychological management (5 items), symptom management (6 items), and compatibility with changes (6 items). In addition, 12 requirements were determined for the app’s technical features. In the next step, a researcher-made questionnaire was designed, which contained three parts (Additional file 1: Appendix 1). Breast cancer patients who were referred to Omid and Imam Khomeini hospitals in Urmia (North West of Iran) for treatment and follow-up received questionnaires from April to June 2020. To analyze the data, we used SPSS (version 16) software. We received 103 responses from breast cancer patients, resulting in a response rate of 85.83%. Additional file 2: Appendix 2 shows our findings in the first phase include demographic and clinical characteristics of the participants, and selected of items related to educational contents and technical features of breast cancer smartphone app (Additional file 2: Appendix 2) [26].

During this phase, we formed an expert team (2 health information technology experts and 2 user experience experts) to design the user interface flow of the self-management app. Balsamiq Mockups software [27] (A low-fidelity wireframing tool that is industry standard) was used to better understand the user experience flow. Mockups have become a popular artifact to capture requirements in sketch of user interface (UI) of the app. In the last few years, its use has been quickly expanded, generating a myriad of tools such as Balsamiq that assistance to create and administrate mockups [28]. The user experience flow of the app was evaluated by 6 patients with breast cancer using the Think aloud method (i.e., Concurrently verbalizing thoughts while working is known as thinking aloud). Used method of thinking aloud to determine their information needs, reasoning in using the computer system, and the source of their problems [29]. Finally, by applying the opinions of the evaluating group, the final version of the user experience flow was considered for app development.

### Second phase

At this phase, we made the development of the mobile app in the Android studio environment. Also, the open-source database management system My Structured Query Language (SQL server), which uses a common computer language and is compatible with Android, was used to developing a database. Our sampling was purposive. Based on previous studies, we could recruit patients [30, 31]. Ultimately, 24 of 103 patients participated in the project. We installed this mobile app for women with breast cancer who were referred to Omid Hospital in Urmia (West Azerbaijan Province, Iran) to follow their treatment. Due to the COVID-19 pandemic and the special conditions of these patients, in the first meeting, their contact information (Email or phone number) was obtained from them so that we could continue to communicate virtually. Inclusion criteria were age between 35 and 60 years, having a diploma or more, and having a smartphone with an Android operating system. Using Zoom software, we organized an online workshop to introduce the self-management app project and provide questionnaires to participants. As part of the workshop, participants learned about the self-management app development project, as well as the project as a whole.

### Third phase

The first step was to provide participants with the QLACS (Quality of Life in Adult Cancer Survivors) questionnaire [32], which they had to complete. This questionnaire consists of 47 questions and the score range is 1–7 (1 = never, 7 = always). We designed the Persian version [33] of the questionnaire QLACS (Validity ratio 0.99) in Google Forum and provided the link (URL) to the participants (*n* = 24). After completing and collecting the questionnaires, we provided the final version of the self-management app to the participants and asked them to use it for three month (1 May to 30 July - 2021). A contact us section was available in the app, where users could ask questions at any time. After the end period (30 July) of using the self-management mobile app, we asked the participants to complete the QLACS questionnaire again. We analyzed data obtained from the QLACS questionnaire using SPSS Statistics 24 (IBM Corp, Armonk, NY, USA). To begin with, descriptive measures were used to report the data on the affecting sections of the generic and specific questionnaires of QLACS, and analysis of paired-sample t-tests was used to measure differences between the main outcomes pre-and post-implementation.

Moreover, for usability evaluation of the app, such as functionality and design; to obtain an overall impression of subjective evaluations of the app, we required participants to answer the SUS (system usability scale) questionnaire [34]. In summary, SUS includes 10 questions rated on the 5-Likert scale (1 = strongly disagree, 5 = strongly agree), this can questionnaire was considered a quantitative example of a qualitative user experience [34–36]. In conclusion, SUS has a proprietary formula, that with its use of it determined the app’s usability in the form of grade and adjective ratings (Table 1) [35].

**Table 1:** Interpreting System Usability Scale (SUS) Score [35]

SUS Score	Grade	Adjective Rating
> 80.3	A	Excellent
68–80.3	B	Good
68	C	Okay
51–68	D	Poor
< 51	F	Awful

## Results

In a cross-sectional descriptive study to identify the desired content for the self-management app for women with breast cancer, we accessed 5 major categories (information acquisition, lifestyle management, psychological management, symptom management, and change compatibility) and 31 sub-categories, most of which directly train self-management skills [26]. According to experts, the following features should be included in the app user experience flow. The self-management app for women with breast cancer should be able to be updated to provide new content to users; if there is a possible problem with the app, it should be resolved in this way. To use the mobile app, users must register (quick and easy registration). Since this mobile app uses a lot of textual content, it must have the functionality to allow users to customize font size, font color, and theme color when reading the text. Content should also have a share button and a bookmark.

**Fig. 2:**
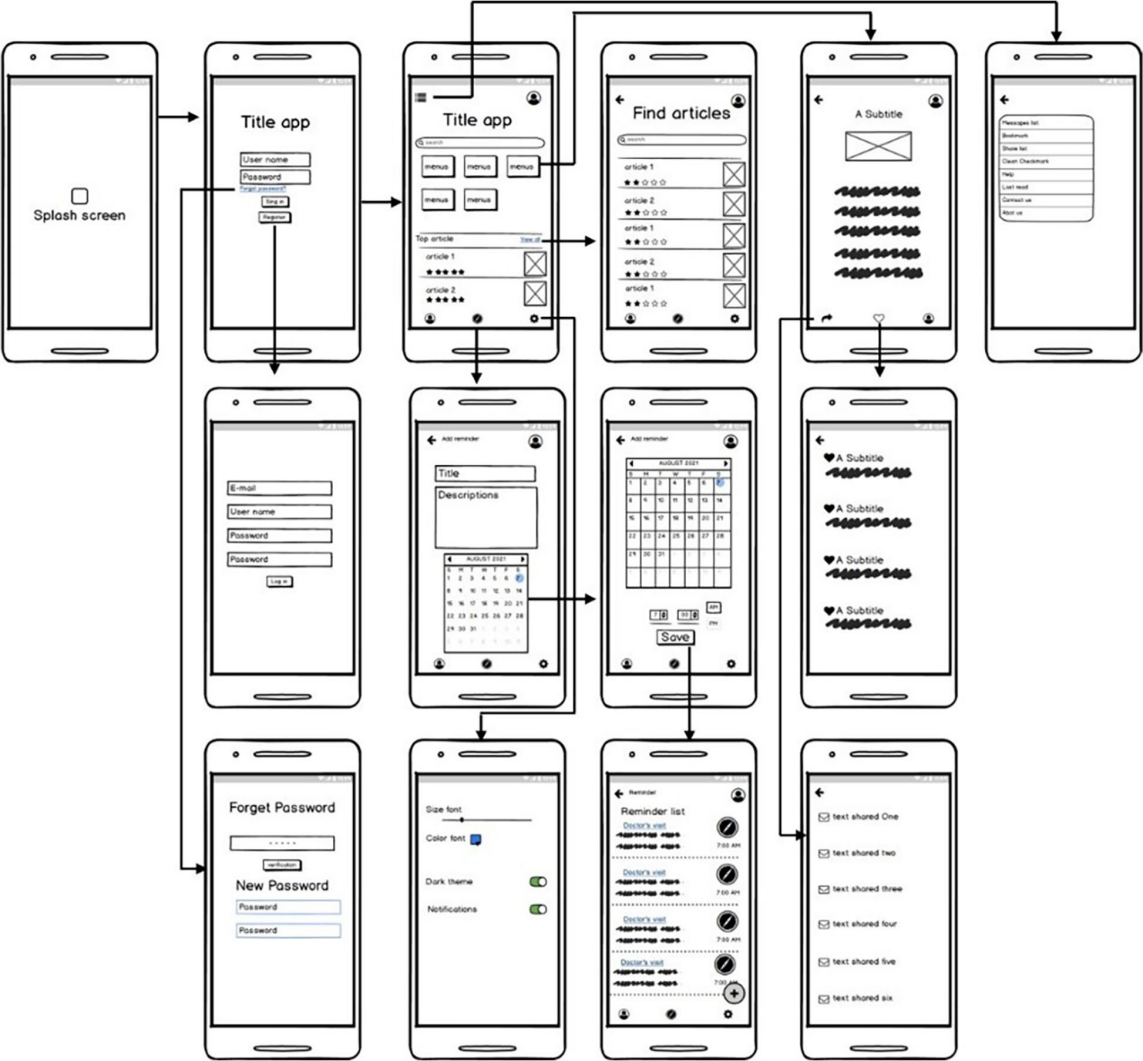
User experience mobile application. In this figure, each of the templates represents the status of an activity. Eacharrow also indicates the direction of the following or previous activity. The figure was created using Balsamiq Mockups software

**Fig. 3:**
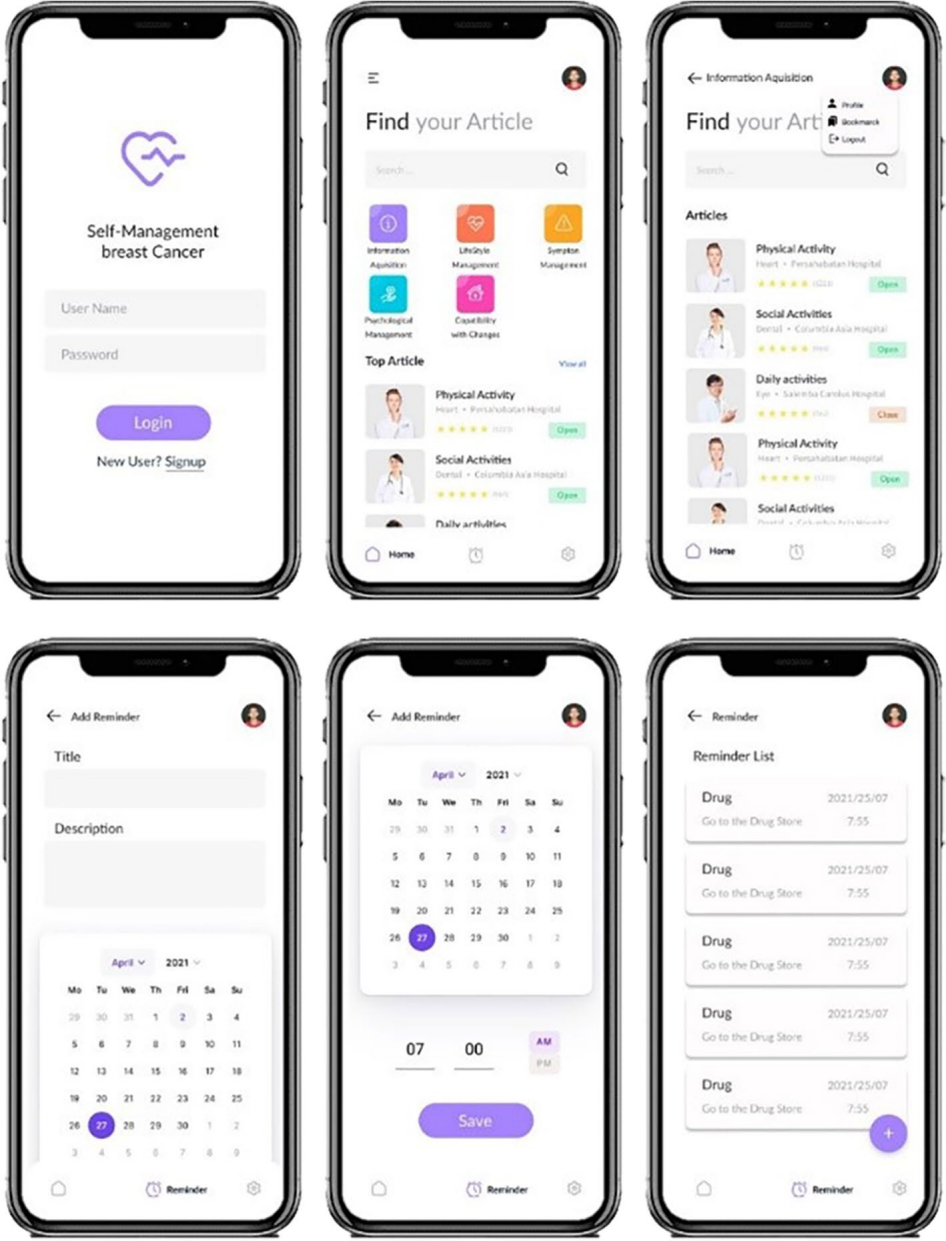
App for self-management of breast cancer patient women, the final version

In this mobile app, there is a section as a reminder that users can set sensitive times (Taking medicine or visiting the doctor). In Fig. 2 shows the user experience flow of the self-management app for women with breast cancer, as well as in Fig. 3, pages of the app version developed in the Android Studio environment.

### Impact rate: pre and post-implementation

As seen in Table 2, 12 (50%) of participants were between the ages of 51–60; The number of participants who were married 21 (87.5%), 12 (50%) had high school diploma degree and also, and 10 (41.66%) of participants have been using a smartphone for 3 to 5.

**Table 2:** Demographics study of participants

Characteristic	Value	N, (%)
**Age**	35–40	4 (16.66%)
	41–50	8 (33.33%)
	51–60	12 (50%)
**Marital status**	Married	21 (87.5%)
	Unmarried	3 (12.5%)
**Education**	High school diploma	12 (50%)
	Some college credits, no degree	4 (16.66%)
	Bachelor’s degree	6 (25%)
	Master’s degree	2 (8.33%)
**Duration of having a smartphone**	1–2	8 (33.33%)
	3–5	10 (41.66%)
	5 - up to	6 (25%)

In the pre-implementation, the participants completed the QLACS questionnaire, and in the post-implementation completed again QLACS in addition to the SUS questionnaire. In section generic (Table 3), the average negative feelings of patients in the pre-implementation was 5.93, thereafter decreasing to 3.40 after the post-implementation. The use of the self-management mobile app caused a reduction in negative feelings. This reduction in negative feelings increases positive feelings in these patients, 2.93 on average in the pre-implementation and increased to 4.66 in the post-implementation. Cognitive problems have an average score of 4.52 after the pre-implementation, which decreases to 3.75 after the post-implementation, which shows the effectiveness of this self-management mobile app. Pain average of 6.37 after the pre-implementation, which drops to a minimum of 4.97 after the post-implementation, which can also be considered a positive effect. In sexual interest, energy/fatigue, and sexual function, there are small reductions after pre-implementation and post-implementation. But social avoidance after the pre-implementation decreases from 6.41 to 3.56, which was a significant decrease. In fact, this reduction reflects the greater impact of the self-management mobile app in this area.

**Table 3:** Analysis of the data obtained from the section generic questionnaire QLACS was carried out using descriptive measures (Mean and SD) and paired-sample t-tests. When *P* < .05 means statistical significance

Generic	Items	Pre		Post		***P*** value
		Mean	SD	Mean	SD	
Negative feelings	You were bothered by mood swings	5.32	0.499	3.33	0.585	<.001
	You felt blue or depressed.	5.11	0.471	3.10	0.72	<.001
	You worried about little things	6.4	0.568	4.03	0.391	<.001
	You felt anxious	6.9	0.422	3.15	0.652	<.001
**Mean**		**5.93**	**0.624**	**3.40**	**0.589**	**<.001**
Positive feelings	You enjoyed life	2.55	0.213	4.23	0.258	<.001
	You were content with your life	2.54	0.213	5.59	0.348	<.001
	You felt happy	3.25	0.258	4.58	0.373	<.001
	You had a positive outlook on life	3.36	0.348	4.23	0.72	<.001
**Mean**		**2.93**	**0.617**	**4.66**	**0.731**	**<.001**
Cognitive problems	You were bothered by having a short attention span	4.25	0.462	4.12	0.462	0.377
	You had trouble remembering things	5.23	0.523	4.36	0.523	<.001
	You had difficulty doing activities that require concentration.	4.25	0.383	3.14	0.383	<.001
	You were bothered by forgetting what you started to do	4.36	0.384	3.36	0.462	<.001
**Mean**		**4.52**	**0.629**	**3.75**	**0.661**	**0.041**
Pain	You were bothered by pain that kept you from doing the things you wanted to do	6.25	0.499	4.52	0.462	<.001
	Your mood was disrupted by pain or its treatment	6.63	0.471	4.36	0.523	<.001
	Pain or its treatment interfered with your social activities	6.25	0.568	5.26	0.383	<.001
	You had aches or pains	6.36	0.422	5.75	0.462	0.001
**Mean**		**6.37**	**0.637**	**4.97**	**0.721**	**<.001**
Sexual interest	You lacked interest in sex	6.45	0.756	5.02	0.568	<.001
	You avoided sexual activity	6.36	0.213	4.48	0.523	<.001
**Mean**		**6.41**	**0.637**	**4.75**	**0.629**	**<.001**
Energy/fatigue	You didn’t have the energy to do the things you wanted to do	6.52	0.348	5.68	0.471	<.001
	You felt tired a lot	6.35	0.373	6.01	0.568	0.017
	You had the energy to do the things you wanted to do	3.05	0.462	4.85	0.422	<.001
	You felt fatigued.	6.52	0.523	5.06	0.471	<.001
**Mean**		**5.61**	**1.570**	**5.40**	**0.740**	**0.010**
Social avoidance	You were dissatisfied with your sex life	6.75	0.568	5.02	0.568	<.001
	You were bothered by being unable to function sexually	6.85	0.5	5.06	0.422	<.001
**Mean**		**6.80**	**0.455**	**5.04**	**0.644**	**<.001**
Social avoidance	You avoided social gatherings	6.36	0.471	4.09	0.523	<.001
	You avoided your friends	6.65	0.568	3.04	0.523	<.001
	You were reluctant to meet new people	6.25	0.422	3.06	0.373	<.001
	You were reluctant to start new relationships	6.36	0.756	4.04	0.462	<.001
**Mean**		**6.41**	**0.653**	**3.56**	**0.718**	**<.001**

According to the cancer-specific section of Table 4, patients’ financial problems increased from 5.50 to 5.99. It is worth noting that the self-management mobile app had no idea about this problem. In benefits, the pre-implementation had an average of 2.74, which increased slightly to 3.12 with a slight increase after the post-implementation, although this increase is very small, but can indicate a change in patients’ attitudes toward their disease after using the self-management mobile app. After the post-implementation and pre-implementation, distress-family had an average of 7, which means that it was registered without any changes; no doubt that this lack of change shows the great importance of this issue. The appearance had a mean of 4.10 after the pre-implementation, which dropped to 3.32 after the post-implementation. Distress-recurrence had a mean of 4.49 after the pre-implementation, which decreased to 4.38 after the post-implementation, this was the lowest decrease (Table 4).

**Table 4:** Analysis of the data obtained from the section cancer-specific questionnaire QLACS was carried out using descriptive measures (Mean and SD) and paired-sample t-tests. When *P* < .05 means statistical significance

Cancer specific	Items	Pre		Post		***P*** value
		Mean	SD	Mean	SD	
Financial problems	You had money problems that arose because you had cancer	6.00	0.373	6.00	0.471	0.664
	You had financial problems due to a loss of income as a result of cancer	6.00	0.462	6.00	0.568	1.000
	You had financial problems because of the cost of cancer surgery or treatment	5.00	0.523	5.60	0.422	<.001
	You had problems with insurance because of cancer	5.00	0.383	6.36	0.756	<.001
**Mean**		**5.50**	**0.677**	**5.99**	**0.550**	**0.416**
Benefits	You felt that cancer helped you to recognize what is important in life	3.20	0.532	4.23	0.471	<.001
	You felt better able to deal with stress because of having had cancer	3.42	0.471	4.25	0.568	<.001
	You realized that having had cancer helps you cope better with problems now	2.09	0.568	2.88	0.422	<.001
	You appreciated life more because of having had cancer	1.20	0.422	1.15	0.756	0.426
**Mean**		**2.47**	**1.010**	**3.12**	**1.340**	**0.107**
Distress-family	You worried about whether your family members might have cancer-causing genes	7.00	0.000	7.00	0.000	0.000
	You worried that your family members were at risk of getting cancer	7.00	0.000	7.00	0.000	0.000
	You worried about whether your family members should have genetic implementations for cancer	7.00	0.000	7.00	0.000	0.000
**Mean**		**7.00**	**0.000**	**7.00**	**0.000**	**0.000**
Appearance	You felt unattractive because of your cancer or its treatment	4.23	0.373	3.52	0.473	<.001
	You were self-conscious about the way you look because of your cancer or its treatment	4.12	0.462	3.24	0.373	<.001
	You felt people treated you differently because of changes to your appearance due to your cancer or its treatment	3.82	0.373	3.12	0.348	0.001
	You were bothered by hair loss from cancer treatment	4.21	0.462	3.40	0.373	<.001
**Mean**		**4.10**	**0.557**	**3.32**	**0.455**	**<.001**
Distress-recurrence	Whenever you felt pain, you worried that it might be cancer again	4.52	0.383	4.08	0.548	0.013
	Whenever you felt pain, you worried that it might be cancer again	4.82	0.348	4.74	0.473	0.257
	You were preoccupied with concerns about cancer	4.12	0.373	4.32	0.462	0.328
**Mean**		**4.49**	**0.575**	**4.38**	**0.591**	**0.205**

**Fig. 4:**
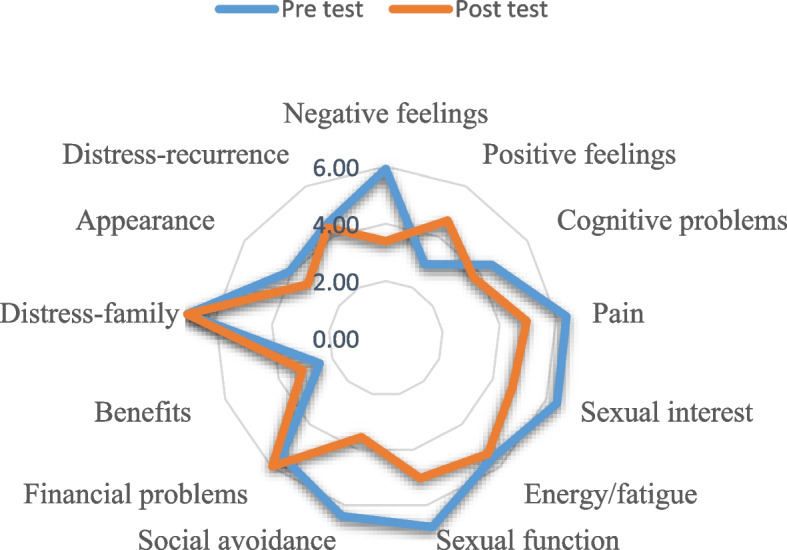
Pre- and post-implementation quality of life for women with breast cancer. The red line shows post-implementation results against pre-implementation results

Figure 4 shows the changes in the quality of life of breast cancer patients after the post-implementation and pre-implementation spider chart.

In line with the findings, the overall usability score of the mobile app for self-management of breast cancer patients women was 82 out of 100 in the evaluation by users. This score is higher than 83 (> 80.3), so based on the SUS formula, we can conclude that such usability of the mobile app is such that: Average SUS score = 83, Adjective Rating = Excellent, and Grade = A.

## Discussion

To instruct self-management skills to women with breast cancer, our app has five main themes: information acquisition, lifestyle management, psychological management, symptom management, and change compatibility [26]. Obviously, to determine the main theme for an education-based app, the main themes for training must first be identified; then a context must be created [37]. Studies similar to ours mostly used the following themes: analyzing side effects, lifestyles, and social activities [38, 39]. In order to design our app user interface to better communicate with patients, we sought out experts in user experience and software in these fields. This method of designing an app’s user interface and user experience flow has been used in a number of studies [40, 41]. It is important to note that a good user interface design is very important to a mobile app’s acceptance by its users [42].

According to our findings, the quality of life of cancer patients was low in some cases after the pre-implementation, but after patients used the self-management app, changes in the quality of life of these patients were observed. These changes are shown in Tables 3 and 4 and also in Fig. 4. The highest level of change was, respectively: social avoidance (pre: 6.41 – post: 3.56), negative feelings (pre: 5.93 - post: 3.40), sexual function (pre: 6.80 - post: 5.04), sexual interest (pre: 6.41 - post: 4.75) and pain (pre: 6.37 - post: 4.97). The cases that received the most impact from the self-management mobile app are definitely appearing among the important ones that affect the quality of life of cancer patients. Many studies have shown that women with breast cancer reduce their presence in the community due to changes in the appearance of their bodies [43, 44]. There are also many other studies on the issue of sexual decline and its function among married women [45, 46]. In our study, 21 of the 24 participants were married. According to our findings, the performance and attractiveness of sexual relations in these patients have improved to a relatively good extent due to educational materials in the mobile app entitled strategies for improving sexuality and teaching auxiliary methods on these issues.

Social avoidance (reluctance to meet and relate to people) and having negative feelings is are very important issues for breast cancer patients. According to previous studies conducted in this field, many solutions to reduce negative emotions (mood swings, feeling anxious, and feeling blue) and encourage participation in the community of these patients have been suggested, including meditation and management of negative emotions [26, 47]. These items were made available to participants in a self-management app (psychological management) in various formats (text, video) [26]. According to our findings, in these patients, there are significant changes in reducing negative emotions and increasing their desire to be in the community compared to the pre-implementation.

Based on the findings in our study the low level of change was, respectively: distress-family (pre: 7 - post:7), distress-recurrence (pre: 4.49 - post: 4.38), benefits (pre: 2.47 - post: 3.12), appearance (pre: 4.10 - post: 3.32). Distress-family did not change during the pre-and post-implementation; all patients with the disease are always concerned about the possibility that their family members may also have cancer and that another family member may be involved. The same anxiety about not improving or returning cancer after recovery has also affected the minds of these patients and has negative effects on their quality of life [48]. Cancer affects the whole life of the affected person and even their partners at a very high level due to physical, financial, mental, and psychological problems. In fact, it is very difficult for a person and their family to accept this disease, so the issue of the benefit of this disease and the appearance affected person (especially if she is a woman) is very difficult and time-consuming for the patient [49–51].

In the third phase and according to our finding obtained through the questionnaire (SUS), which was analyzed based on the special formula of SUS, wecalculated that the overall rating for mobile app for self-management of women with breast cancer leads to a SUS score equal to 83 (out of 100), which is considered an excellent level (> 80.3), and grade A. This usability evaluation shows that users well considered the app in terms of functionality and design. According to app evaluation studies, a mobile app has the most and best impact when it is designed at a standard and appropriate level in terms of functionality. Undoubtedly, this issue makes the developed m-health app more used by users [42, 52].

The self-management mobile app in the generic section had a greater impact on quality of life than those in the cancer-specific section. As shown in the results, the self-management mobile app orients to general topics and content in order to increase the quality of life for breast cancer patients. We believe that the presence of both IOS and Android versions along with a larger number of patients will produce more accurate results in this study. But this was our limitation and we were unable to do so due to a lack of financial resources and lack of a dedicated budget for this project. We were also limited by the fact that our study population consisted of women from only one province (Urmia) in Iran, and we were unable to consider patients (women with cancer) from other cities because of low budget. Even though our study city had a large population and met our needs, a broader study across the country and with two versions IOS and Android could produce more desirable and broader results. This might be a future research project.

## Conclusion

This study demonstrates the potential of a breast cancer self-management app to support and improved the quality of life for women with breast cancer. We conducted this study to show that by developing a self-management app, women with breast cancer can improve their quality of life by increasing their self-management skills. However, we considered only Iranian women in this study because such a mobile app aimed at the self-management of Iranian women with breast cancer had not been developed before. For this reason, it may not be reasonable to generalize the usefulness of this mobile app to all women with breast cancer.

## Supplementary Information

**Additional file 1.**

**Additional file 2.**

## Data Availability

All data and information created or examined during this study are contained in this published article.

## Abbreviations

App: Application
QLACS: Quality of Life in Adult Cancer Survivors
IOS: iPhone OS
URL: Uniform Resource Locator
