# Supplementary material for: Effectiveness of a self-management mobile app on the quality of life of women with breast cancer: a study in a developing country
Source: BMC Womens Health. 2022 Nov 11;22:446. doi: 10.1186/s12905-022-02020-5 (PMC9652046; doi:10.1186/s12905-022-02020-5)
Supplement: Supplementary file 1 — Additional file 1. [file 12905_2022_2020_MOESM1_ESM.docx]

| **Part 1:Demographic and clinical characteristics of the Participants** | |
| --- | --- |
|  | 1. Age in years: |
| Married □ Single □ widowed □ Divorced □ | 1. Marital status: |
| Urban □ Rural □ | 1. Place of residence |
| Less than one year □ 1 to 3 years □ 3 to 5 years □ More than 5 years □ | 1. How long have you been suffering from breast cancer? |
| Left breast □ Right breast □ Both breasts□ | 1. On which side of the body is the disease present? |
| Surgery □ Chemotherapy □ Radiotherapy □ Hormone therapy □ All □ None □ | 1. What is your type of treatment? |
| Mastectomy of one breast □ Mastectomy of both breasts □ Lumpectomy of one breast □ Lumpectomy of both breasts□ | 1. If you undergo surgery, choose its type? |
| Yes □ no □ | 1. Do you have a history of breast cancer in your family? |
| No activity □ 1-3 hours □ 4-7 hours □ More than 7 hour □ | 1. How much is your physical activity (in hours per week)? |
| Yes □ no □ | 1. Do you use a personal smart phone? |
| Yes □ no □ | 1. In the past six months, have you used your phone to access general health information related to breast cancer? |
| Yes □ no □ | 1. Do you want to use your phone to track cancer information through a smart phone application? |

**Appendix 1**

| **Part2: Please indicate the importance of each of your educational needs.** | | | | | | |
| --- | --- | --- | --- | --- | --- | --- |
| How much do you agree to receive information about the content below? | | Strongly agree | Agree | So so | Disagree | Strongly disagree |
| 1 | Breast anatomy | 5 | 4 | 3 | 2 | 1 |
| 2 | Types of breast cancer | 5 | 4 | 3 | 2 | 1 |
| 3 | Different types of breast cancer treatments | 5 | 4 | 3 | 2 | 1 |
| 4 | Common side effects of breast cancer treatment | 5 | 4 | 3 | 2 | 1 |
| 5 | Breast surgery | 5 | 4 | 3 | 2 | 1 |
| 6 | Reconstructive breast surgery | 5 | 4 | 3 | 2 | 1 |
| 7 | Effects of the disease on quality of life | 5 | 4 | 3 | 2 | 1 |
| 8 | Physical activity(Exercising and walking) | 5 | 4 | 3 | 2 | 1 |
| 9 | Dietary modifications | 5 | 4 | 3 | 2 | 1 |
| 10 | Pregnancy after treatment | 5 | 4 | 3 | 2 | 1 |
| 11 | Daily activities | 5 | 4 | 3 | 2 | 1 |
| 12 | Social activities | 5 | 4 | 3 | 2 | 1 |
| 13 | Physical health | 5 | 4 | 3 | 2 | 1 |
| 14 | Sexual health | 5 | 4 | 3 | 2 | 1 |
| 15 | Managing negative emotions (anxiety and depression) before/after disease | 5 | 4 | 3 | 2 | 1 |
| 16 | Necessary skills to manage the effects of illness on patients' morale | 5 | 4 | 3 | 2 | 1 |
| 17 | Stressors during disease | 5 | 4 | 3 | 2 | 1 |
| 18 | Fear of recurrence | 5 | 4 | 3 | 2 | 1 |
| 19 | Relaxing | 5 | 4 | 3 | 2 | 1 |
| 20 | Impact of spirituality | 5 | 4 | 3 | 2 | 1 |
| 21 | Pain management and ways to reduce it | 5 | 4 | 3 | 2 | 1 |
| 22 | Management of chemotherapy side effects | 5 | 4 | 3 | 2 | 1 |
| 23 | Management of radiotherapy side effects | 5 | 4 | 3 | 2 | 1 |
| 24 | Management of surgery side effects | 5 | 4 | 3 | 2 | 1 |
| 25 | Stress management | 5 | 4 | 3 | 2 | 1 |
| 26 | Acceptance of disease | 5 | 4 | 3 | 2 | 1 |
| 27 | Disease compatibility | 5 | 4 | 3 | 2 | 1 |
| 28 | Finding new fun activities | 5 | 4 | 3 | 2 | 1 |
| 29 | Empowering self-care | 5 | 4 | 3 | 2 | 1 |
| 30 | Adaptation to physical changes | 5 | 4 | 3 | 2 | 1 |
| 31 | Adaptation to emotional problems | 5 | 4 | 3 | 2 | 1 |
| 32 | Creating a sense of purpose | 5 | 4 | 3 | 2 | 1 |
| **Part 3: Please specify the level of interest and tendency to use each of the features of the application. If you think of other cases, mention them.** | | | | | | |
| How much do you agree with the use of the technical features mentioned below? | | Strongly agree | Agree | So so | Disagree | Strongly disagree |
| 1 | Ease of use of app | 5 | 4 | 3 | 2 | 1 |
| 2 | Simple and well-ordered visual interface | 5 | 4 | 3 | 2 | 1 |
| 3 | Security and privacy of patient information | 5 | 4 | 3 | 2 | 1 |
| 4 | Reminders about drug, diet, exercise, appointment | 5 | 4 | 3 | 2 | 1 |
| 5 | Ability to communicate with the health team | 5 | 4 | 3 | 2 | 1 |
| 6 | Up- to-date and supportive services of app | 5 | 4 | 3 | 2 | 1 |
| 7 | Ability to share experiences with other patients via chat | 5 | 4 | 3 | 2 | 1 |
| 8 | Consistency of elements and icons | 5 | 4 | 3 | 2 | 1 |
| 9 | Fast loading screens | 5 | 4 | 3 | 2 | 1 |
