# Supplementary material for: Effectiveness of a self-management mobile app on the quality of life of women with breast cancer: a study in a developing country
Source: BMC Womens Health. 2022 Nov 11;22:446. doi: 10.1186/s12905-022-02020-5 (PMC9652046; doi:10.1186/s12905-022-02020-5)
Supplement: Supplementary file 2 — Additional file 2. [file 12905_2022_2020_MOESM2_ESM.docx]

**Appendix 2**

**Table 1. Demographic and clinical characteristics of the Participants**

| **Variables** | **Value** |
| --- | --- |
| **Age in years** | N (%) |
| < 30 | 4 (3.88%) |
| 30-40 | 20 (19.42%) |
| 40-50 | 42(40.78%) |
| 50-60 | 25 (24.27%) |
| ≥ 60 | 12(11.65%) |
| **Marital statues** | N (%) |
| Married | 68 (66.02%) |
| Single | 16 (15.53%) |
| widowed | 13 (12.62%) |
| Divorced | 6 (5.83%) |
| **Education level** | N (%) |
| University degree | 26 (25.24%) |
| High school(diploma) | 48 (46.60%) |
| Primary school | 21 (20.39%) |
| Illiterate | 8 (7.77%) |
| **Work status** | N (%) |
| Unemployed | 66 (64.08%) |
| Employed | 37 (35.92%) |
| **Treatment modality** | N (%) |
| Chemotherapy | 103 (100%) |
| Surgery | 85 (82.52%) |
| Radiation therapy | 53(51.45%) |
| Hormone therapy | 37 (35.92%) |
| **Place of residence** | N (%) |
| Urban | 76 (73.79%) |
| Rural | 27 (26.21%) |
| **Family history of breast cancer** | N (%) |
| Yes | 32 (31.07%) |
| No | 71 (68.93%) |
| **Use of personal smartphone** | N (%) |
| Yes | 91 (88.35%) |
| No | 12 (11.65%) |
| **Interested in using  breast cancer applications** | N (%) |
| Yes | 88 (85.43%) |
| No | 15 (14.57%) |

**Table2. Mean of items related to educational contents of breast cancer smartphone app (range 1-5)**

| **desired features**        **Information acquisition** | **Items** | **Mean** | **S.D** |
| --- | --- | --- | --- |
|  | common side effects of treatment | 4.68 | 0.51 |
|  | effects of the disease on quality of life | 4.33 | 1.05 |
|  | different types of treatments | 4.17 | 0.80 |
|  | reconstructive breast surgery | 4.14 | 1.07 |
|  | types of breast cancer | 4.05 | 1.02 |
|  | breast surgery | 4.05 | 1.02 |
|  | breast anatomy | 3.48 | 1.23 |
|  | physical activity | 4.55 | 0.68 |
|  | dietary modifications | 4.42 | 0.73 |
|  | social activities | 4.14 | 0.91 |
| **Lifestyle management** | physical health | 3.68 | 1.01 |
|  | daily activities | 3.43 | 1.14 |
|  | sexual health | 3.37 | 1.11 |
|  | Pregnancy | 3.28 | 1.34 |
|  | impact of spirituality | 4.15 | 0.97 |
|  | stressors during disease | 4.11 | 0.79 |
| **Psychological management** | relaxing | 3.98 | 0.99 |
|  | fear of  recurrence | 3.95 | 0.95 |
|  | management of negative emotions | 3.86 | 1.08 |
|  | management of chemotherapy side effects | 4.45 | 0.78 |
|  | pain management | 4.33 | 0.81 |
| **Symptom management** | stress management | 4.25 | 0.87 |
|  | management of surgery side effects | 4.08 | 0.99 |
|  | management of radiotherapy side effects | 3.90 | 1.17 |
|  | empowering self-care | 4.03 | 0.66 |
|  | adaptation to physical changes | 4.18 | 1.12 |
|  | finding new fun activities | 4.05 | 0.91 |
| **Compatibility with changes** | adaptation to emotional problems | 3.92 | 0.92 |
|  | acceptance of disease | 3.73 | 1.01 |
|  | disease compatibility | 3.55 | 0.96 |
|  | creating a sense of purpose | 3.37 | 1.15 |

**Table3. Technical features of breast cancer smartphone app (range 1-5)**

| **Ease of use of app** | 4.83 | 0.42 |
| --- | --- | --- |
| **Simple and well-ordered visual interface** | 4.75 | 0.48 |
| **Security and privacy of patient information** | 4.63 | 0.55 |
| **Reminders about drug, diet, exercise, appointment** | 4.55 | 0.62 |
| **Ability to communicate with the health team** | 4.42 | 0.73 |
| **Up- to-date and supportive services of app** | 4.24 | 0.94 |
| **Ability to share experiences with other patients via chat** | 4.20 | 0.88 |
| **User customization** | 4.07 | 1.06 |
| **Appropriate use of color , texture, font, and graph** | 3.93 | 1.11 |
| **Consistency of elements and icons** | 3.88 | 1.18 |
| **Fast loading screens** | 3.67 | 1.22 |
| **Large Touch and easy Navigation** | 3.59 | 1.35 |
